# Estimating Time of Coordinate Measurements Based on the Adopted Measurement Strategy

**DOI:** 10.3390/s22197310

**Published:** 2022-09-27

**Authors:** Marek Magdziak

**Affiliations:** Department of Manufacturing Techniques and Automation, Faculty of Mechanical Engineering and Aeronautics, Rzeszów University of Technology, Al. Powstańców Warszawy 12, 35-959 Rzeszów, Poland; marekm@prz.edu.pl; Tel.: +48-178651491

**Keywords:** coordinate measuring technique, measurement strategy, time of coordinate measurements, form deviation, flatness deviation

## Abstract

This article concerns coordinate metrology and contact coordinate measurements which are conducted using a coordinate measuring machine. The paper presents the results of experimental investigations, based on which a model to calculate the time needed to conduct coordinate measurements to determine the flatness deviation of a selected surface of a machined product was developed. The time taken and the accuracy of the coordinate measurements influence the effectiveness of coordinate measurements of geometrical quantities of products and the productivity of the entire manufacturing process. Therefore, when planning a strategy for the coordinate measurement process, apart from considering the measurement uncertainty, the duration of individual measurement tasks should also be taken into account. A coordinate measurement time model was developed by use of response surface methodology (RSM). Experimental research was carried out using an ACCURA II coordinate measuring machine equipped with a VAST XXT measuring probe and cooperating with Calypso inspection software. The coordinate measurement time model which was developed was implemented in the selected metrology software of the coordinate measuring machine. The purpose of the implementation was to enable application of the mathematical model created into industrial practice.

## 1. Introduction

The coordinate measuring technique is widely used in various branches of industry, such as the aerospace and automotive industries, for coordinate measurements of products which are characterised by a regular geometric shape and composed of curvilinear surfaces [1,2]. Coordinate measurements of products can be made with the use of various contact and non-contact coordinate measuring systems [3,4,5], which include, for example, coordinate measuring machines, coordinate measuring arms, computed tomography systems, measuring systems based on photogrammetry and CNC machine tools equipped with measuring probes [4,6]. Multisensory systems are also very popular. Ren et al. [7] combined the advantages of contact and non-contact measuring systems to accurately reconstruct 3D surfaces. A comparison of the results of measurements carried out using contact and non-contact measuring systems is presented in [8]. The selection of a measuring system depends, among other things, on the expected measurement accuracy. The most accurate coordinate measuring systems are coordinate measuring machines (CMMs), which today can be equipped with both contact and non-contact measuring probes. Contact measurements are still often used in industrial practice [7] and can be carried out in both scanning and single-point probing modes [5]. Multi-axis scanning of curvilinear surfaces is presented in [9], and this also includes reporting the algorithm for generating the movement path of a measuring probe. In addition, a comparison of measuring probes enabling single-point probing and scanning modes, taking into account the measurement uncertainty and the time of measurements, is presented in [10]. Coordinate measurements conducted in a scanning mode are more advantageous than measurements performed in a single-point probing mode, due to the shorter duration of a given measurement task.

**Figure 1:**
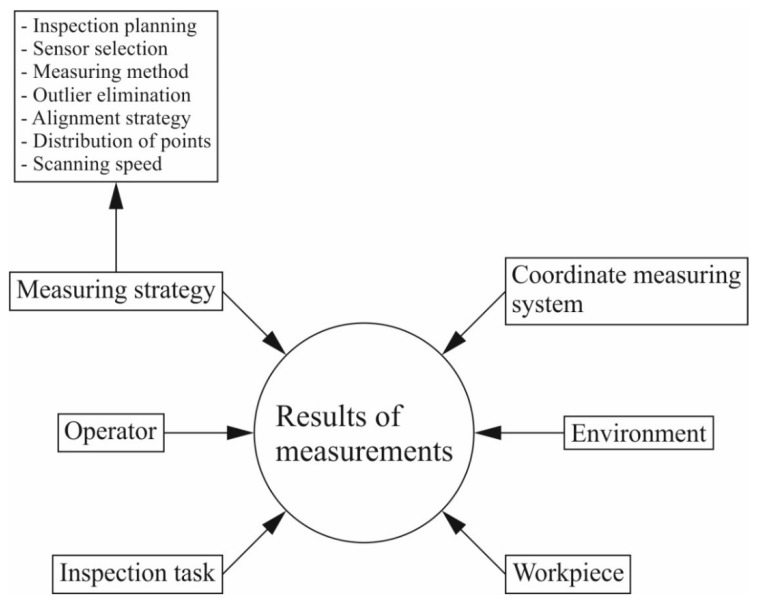
Review of factors influencing results of coordinate measurements [13].

This article concerns coordinate measurements carried out by means of coordinate measuring machines cooperating with contact scanning measuring probes, which produce more accurate measurement data than CMMs with non-contact measuring probes [5]. The effectiveness of contact coordinate measurements depends on the accuracy of the measurements and the duration of a given measurement task. The accuracy of contact coordinate measurements is influenced by a number of factors, including for instance, the environmental conditions prevailing in a measurement laboratory where a coordinate measuring machine is located, the accuracy of the coordinate measuring system and the applied measurement strategy [11]. Depending on the type of a coordinate measuring system, a measurement strategy may include various elements. For example, in the case of a coordinate measuring machine equipped with a contact measuring probe, the elements of a measurement strategy may include the scanning speed, the number of measurement points and their distribution on the surface of a measured product, the probe radius correction method adopted for calculating corrected measurement points based on the indicated measurement points, the filtration parameters of the measurement results, the methods of calculating associated elements, the ways of mounting a measured product on a CMM and determining a coordinate system of the workpiece under investigation, the settings regarding the removal of outliers and the use of a rotary table [11,12,13]. An overview of the factors determining the final coordinate measurement results is presented in Figure 1, which also includes examples of the elements that are part of the coordinate measurement strategy. The measurement strategy is one of the factors that have the greatest influence on the contact coordinate measurement results [13]. Therefore, in order to carry out accurate and fast coordinate measurements on a selected product, it is necessary not only to select the right measuring system, but also to use a measurement strategy appropriately selected for the given measurement task [14].

A number of research articles have been published concerning the various elements of a measurement strategy; e.g., the distribution of measurement points on the investigated surfaces of a measured object. The location of the measurement points on measured objects is very important when assessing the quality of a product. Scientists have proposed a number of strategies to select the best distribution of measurement points to speed up a measurement process [15]. However, metrologists still independently decide the distribution of measurement points based on both their experience and intuition [16]. Li et al. [1] presented the enhanced maximin distance method, dedicated mainly to non-contact measurements, which is based on normal vectors calculated at measurement points. The method was applied to the selection of measurement points in order to obtain a simpler point cloud. Moroni and Petrò [14] proposed the model of costs of measurements, which can be used to choose the distance between measurement points. Ren et al. [2] presented an algorithm for distributing the paths on a curvilinear surface along which a scanning process is conducted. The position of a scanning line is obtained based on an analysis of the complexity of the surface under investigation and the deviation between a substitute surface and its nominal model. Moreover, [17] presented a method for selecting measurement points for non-contact measurements of curvilinear surfaces. Measurements are conducted along cross-sections calculated between the surface under consideration and a set of planes. Lalehpour et al. [18] investigated three methods of distributing the measurement points in order to select the best one. The authors of [19] presented a method for selecting points on a blade. New methods to determine the distribution of measurement points, based on the lengths of curves and the areas of surfaces, were proposed in [20,21].

Additionally, Wang et al. [22] compared selected methods for determining the distribution of points, including a uniform distribution, for three surfaces. Pagani and Scott [23] presented a strategy for distributing points based on the length of a curve and its complexity, and compared it to other methods. In turn, the article [24] proposed a method for determining a measurement path in order to carry out measurements on a CNC machine tool. The measurement results were compared to the results of coordinate measurements obtained using a CMM. The method created was based on a CAD model of an analysed object. Mian et al. [16] analysed various strategies for the distribution of measurement points in the assessment of flatness deviation, and their influence on the time required to obtain the coordinate measurements, in order to determine the efficiency of the algorithms under consideration.

The authors of [15] proposed a new measurement strategy to improve the efficiency of the evaluation of machining errors through the use of coordinate measuring machines. In the first stage, the measurement results are obtained based on a sparse distribution of measurement points in order to reduce measurement time. Then, by using a neural network, machining errors are obtained that correspond to a dense distribution of measurement points.

Moreover, the authors of [25,26] analysed the influence of an adopted measurement strategy on the coordinate measurement results used to determine flatness and cylindricity deviations. They considered the use of different styli and various densities of measurement points. The authors of [27] also analysed the influence of, among other factors, the diameter of a stylus tip on the results of the measurements of a gauge ring. The article [27] takes into account other elements of a measurement strategy, such as the scanning speed. The influence of the number of measurement points on coordinate measurement results for free-form surfaces was also analysed in [28]. The authors of [28] found that the use of 25 points located along cross-sections of a blade is sufficient, and significantly reduces measurement time. On the other hand, the authors of [29] proposed a two-stage algorithm for generating a movement path for a measuring probe in the case of five-axis scanning of a product’s surface, which ensures a collision-free measurement. The algorithm was based on image analysis to determine the acceptable area for a stylus. Moreover, a strategy for generating the path of a measuring probe on the basis of an improved ant colony algorithm, which may increase the efficiency of coordinate measuring machines, was presented in [30].

The strategy used for coordinate measurement may also include various methods of defining the coordinate system of a measured workpiece. The article [31] presents the influence of errors on the results of the measurement of a gear when determining a coordinate system. The selection of parameters for the filtration of the measurement points is also an element of a measurement strategy. The authors of [32] presented a method of reducing measurement noise in the case of non-contact measurements of the flat surfaces of products. In the coordinate measuring technique, the probe radius correction process has a significant impact on the coordinate measurement results [33]. There are many works, e.g., [34,35], dealing with the problem of the probe radius correction process.

The elements of the measurement strategy selected also affect the duration of the coordinate measurement process, which is the part of the overall time of a product manufacturing process. In the case of a CMM cooperating with a scanning contact measuring probe, the measurement time can be modified by changing the speed of coordinate measurement carried out in the scanning mode, and by modifying the measurement path. In contrast, during measurements taken in a single-point probing mode, the time of the measurement process mainly depends on the distribution of the points on the surface of the measured product, and the speed of the non-measuring movements of the measuring probe of the CMM. The distribution of the points may result, in turn, from the declared distance between the measurement points. In order to carry out effective coordinate measurements, it is necessary to carefully select a measurement strategy that will ensure both high measurement accuracy and the shortest possible time for coordinate measurement.

Unfortunately, commercial metrology software that cooperates, for example, with coordinate measuring machines, does not enable the verification of the duration of the coordinate measurement process based on the adopted parameters for the product measurements before it starts. This, in turn, makes it much more difficult to choose the right measurement strategy to enable the implementation of effective coordinate measurement. Therefore, it is necessary to supplement the capabilities of commercial metrology software packages that are part of modern coordinate measuring systems. This is made possible by using, for example, parametric programming of coordinate measurements. Parametric programming enables the implementation of mathematical models, on the basis of which, for instance, the results and times of coordinate measurements can be estimated for the commercial inspection software used in industry. An example of metrology software that enables parametric programming is the Calypso software developed by the Carl Zeiss company (Oberkochen, Germany). The parametric method of programming within the Calypso software is possible thanks to its PCM module (parameter coded measurements).

Mathematical models that can be used in metrology software cooperating with, e.g., coordinate measuring machines, can be developed using response surface methodology (RSM). This methodology, among others, has been widely used in the metrology of geometrical quantities, in coordinate measuring techniques and in mechanical engineering [36,37]. For example, the authors of [36] applied RSM to develop mathematical models, on the basis of which it was possible to estimate the quality of virtual machining of curvilinear surfaces of a product, as well as to determine the accuracy and duration of coordinate measurement processes. However, the measurement time was not estimated on the basis of the results of real coordinate measurements. The results, on the basis of which the mathematical models were developed, were obtained by means of computer simulations of manufacturing and measurement processes. Moreover, the measurement time was determined based only on the length of the measurement path. The application of RSM in the case of machining of Ti6Al4V titanium alloy was presented in [37]. Mathematical models of the temperature in a cutting zone and two components of a cutting force were developed. The input parameters were as follows: cutting speed, depth of cut and feed.

This paper presents the possibility of using RSM to develop a mathematical model of the time needed to undertake contact coordinate measurements to determine the flatness deviation of a selected surface of a product after its machining. Moreover, special attention was paid to the method of implementation of the model in the selected metrological software, to increase the possibility of applying the mathematical model in industrial practice. Thus, the user of a coordinate measuring machine will be able to estimate how the adopted coordinate measurement parameters will translate into the time needed to conduct a given measurement task. The advantage of the model presented is the fact that it was developed on the basis of the results of actual coordinate measurements of a selected product.

The following parts of this article concern, inter alia, descriptions of the experimental investigations performed, the development of the model for determining the coordinate measurement time and the implementation of the model in the Calypso software from the Carl Zeiss company. The article ends with the conclusions drawn on the basis of the results of the experimental research.

## 2. Materials and Methods

### 2.1. Experimental Investigations

**Figure 2:**
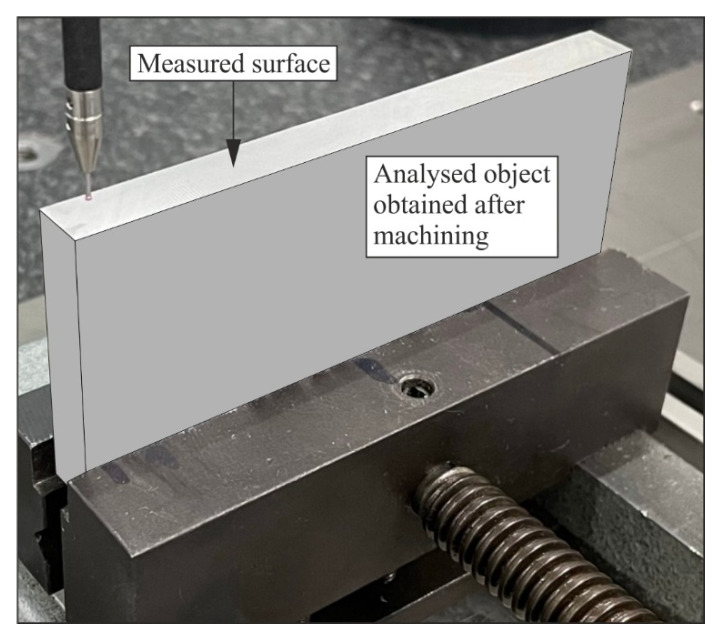
The sample upon which coordinate measurements to determine the flatness deviation of the upper surface were performed.

A model to calculate the contact coordinate measurement time required to determine the flatness deviation of a selected surface of a measured product was developed within the following work. A series of coordinate measurements of a sample obtained as a result of machining conducted by milling were made in order to develop the model (Figure 2). The machining was performed using a DMU 100 monoBLOCK multi-axis machine tool. The experimental research was conducted in accordance with the adopted research plan.

**Figure 3:**
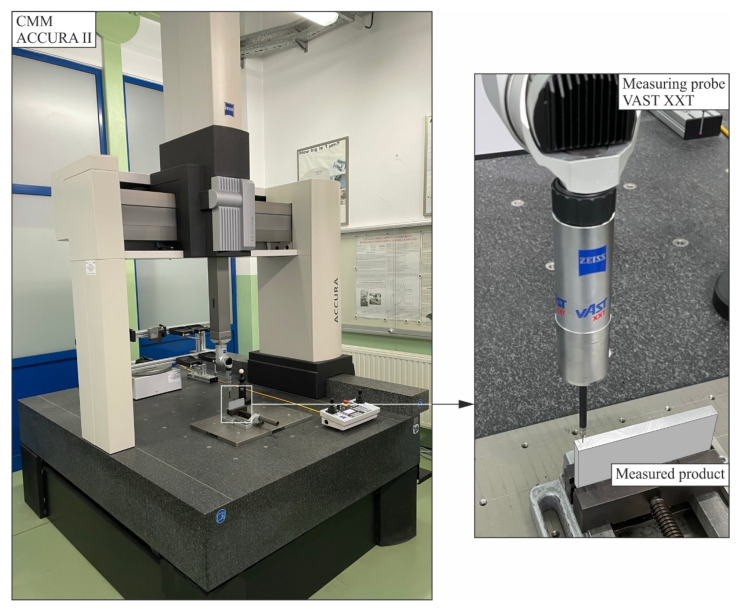
The ACCURA II coordinate measuring machine equipped with the VAST XXT measuring probe, which was used to measure the flatness deviation of the selected surface.

The coordinate measurements of the object under consideration were conducted using an ACCURA II coordinate measuring machine equipped with a VAST XXT contact scanning probe. The coordinate measuring system applied is presented in Figure 3. The coordinate measuring machine was characterised by the following accuracy parameters [12]:

1. *E_MPE_* = (1.6 + *L*/333) μm;
2. *P_MPE_* = 1.7 μm;
3. *T_MPE_* = 2.5 μm;
4. *τ_MPT_* = 50.0 s.

where *E_MPE_* is the maximum permissible error of a length measurement; *L* is the measured length in mm; *P_MPE_* is the maximum permissible single-stylus form error; *T_MPE_* is the maximum permissible scanning error; and *τ_MPT_* is the maximum permissible scanning test duration.

The flatness deviation was calculated by using the functions of the Calypso software cooperating with the above-mentioned coordinate measuring system. The minimum element, which is the properly chosen associated element when assessing form deviations in the coordinate measuring technique, was used in the applied inspection software. Measurement results were not filtered and outliers were not removed. The flatness deviation was measured using various measurement strategies determined within the Plane measurement element, which is available in the Calypso software cooperating with the CMM ACCURA II.

### 2.2. Measurement Strategies Considered

**Figure 4:**
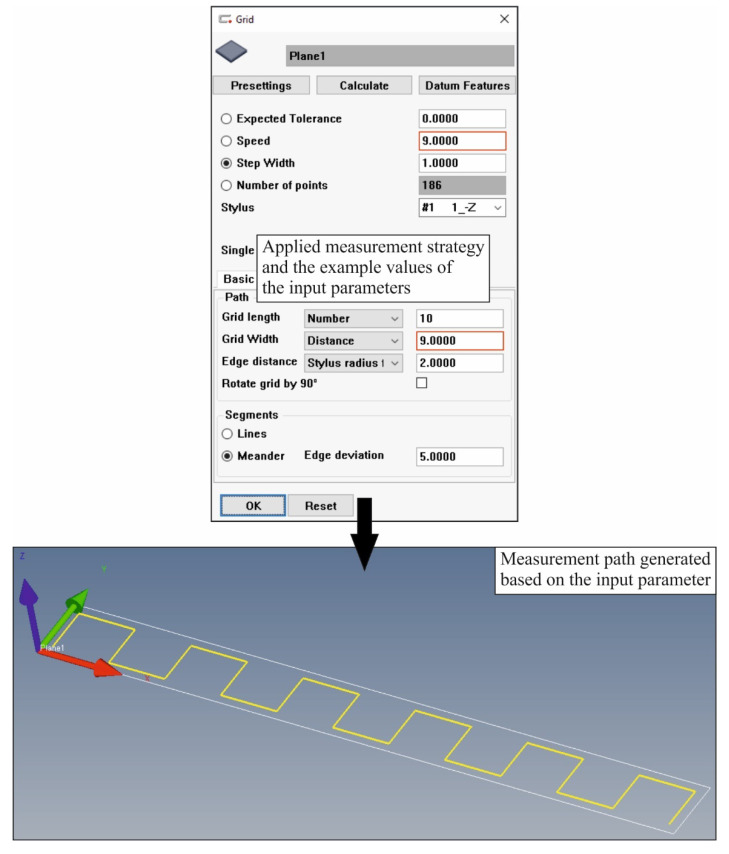
An example of a measurement strategy used during experimental investigations and the method of determining the values of parameters of contact coordinate measurements to determine a flatness deviation.

Measurements of the flatness deviation of the selected surface of the product were carried out using various measurement strategies, which were defined by modifying the scanning speed and the distance between measurement paths. The other measurement parameters, i.e., the distance between measurement points and the distance of the measurement points from the boundaries of the measured surface, were not modified; these values were the same for all measurement strategies under consideration. Therefore, they had no influence on the differences between the measurement results that were obtained with the analysed measurement strategies. The sampling step applied was equal to 1.0 mm, and the distance of points from the surface boundaries was equal to 2.0 mm. The value of the sampling step, i.e., the distance between the measurement points measured in the scanning mode, was based on the values compliant with the recommendations of the manufacturer of the applied coordinate measuring system, i.e., the Carl Zeiss company. These recommendations were published in [38]. The final number of measurement points located on the surface of the measured product for the assumed measurement path of the measuring probe, based on which the flatness deviation was calculated, resulted from the distance between the measurement paths and the sampling step. Figure 4 shows an example of the measurement strategy applied during the experimental research.

This figure also presents the Calypso software dialog box that enables the definition of different measurement strategies. The figure shows the coordinate measurement parameters, the modification of which made it possible to create the measurement strategies under consideration. The analysed parameters which mainly influenced the time of coordinate measurements were the scanning speed and the distance between measurement paths (grid width).

Moreover, the shape of the measurement path of the measuring probe was not modified during the experimental tests, and the path shown in Figure 4 was applied. The advantage of the selected path of the probe is that it includes many measurement points uniformly distributed along the measured surface of the analysed product. This measurement path increases the probability of identifying the maximum local deviations that determine the value of a flatness deviation. The chances of detecting the maximum local deviations decrease when using other types of paths (e.g., measurement paths based on a polyline or a circle, which are also available in the Calypso software).

### 2.3. Response Surface Methodology

**Figure 5:**
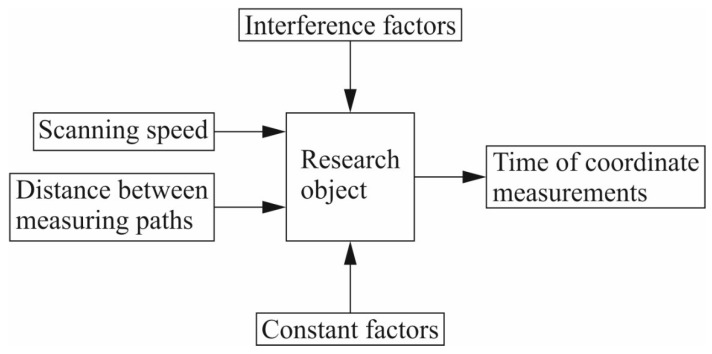
A functional diagram of the measurement process, taking into account the input factors.

The model to calculate the coordinate measurement time was developed, as mentioned before, using RSM. The input factors were the scanning speed and the distance between the measurement paths along which the scanning process of the measured surface of the product was carried out. The output factor was the coordinate measurement time. The constant factors were, among others, the sampling step, the distance of the measurement path of the probe from the boundaries of the measured surface, the selected measuring probe stylus and the calculation method for an associated element. The interference factors were, for example, the vibrations and environmental conditions prevailing in the measurement laboratory where the ACCURA II coordinate measuring machine was located (Figure 5).

The advantage of RSM is that it is easy to use under industrial conditions, and thus in the coordinate measuring technique. In the case of the research conducted, long-term coordinate measurements were not required, so could be used by the employees of quality control departments. The experimental research plan and the results of the statistical analysis were generated automatically by means of statistical software. In addition, the research plan could also be easily implemented in commercial measurement software cooperating with coordinate measuring systems, which could lead to the automation of experimental tests based on coordinate measurements. Therefore, if it is necessary, for example, to modify the path of a measuring probe (as a result of e.g., changing the shape of a measured product), the model of coordinate measurement time can be relatively quickly updated.

## 3. Results of Experimental Investigations

The experimental studies were carried out using a central composite design according to the data presented in Table 1. In addition, Table 1 presents the assumed distances between the measurement paths and the applied scanning speeds, by means of which the various strategies to make the contact measurements needed to calculate the flatness deviation were determined. The extreme values of the input parameters were partly selected based on [38]. In Table 1, the first three columns are as follows: StdOrder—non-random order of experimental tests; RunOrder—random order of experimental tests; and PtType—a point type that specifies various combinations of values of input parameters.

The times for the coordinate measurement processes were recorded for each experimental test, and are presented in Table 2.

The results obtained from the experimental research were used to develop the model for the coordinate measurement time needed to determine a flatness deviation. Analysis of variance (ANOVA) was performed on the basis of the results obtained during the contact coordinate measurements. The statistical analysis of the results of the experimental tests was performed using Minitab software. The proposed model of measurement time *t* as a function of the distance between measurement paths *d* and the scanning speed *v* is presented as Equation (1). A linear + squares model was developed. The coefficient of determination of the created model was equal to 99.42%.

−*t*^−0.5^ = −0.00645 − 0.01232·*d* − 0.01841·*v* + 0.000449·*d*·*d* + 0.000482·*v*·*v* (1)

The results of the analysis of variance in relation to individual elements of the coordinate measurement time model are presented in Table 3, in which the columns DF, Adj SS, Adj MS are the total degrees of freedom, adjusted sums of squares and adjusted mean squares, respectively. Adj SS and Adj MS were used to calculate the *p*-Value for a term and the coefficient of determination. In turn, the F-Value was used to calculate the *p*-Value, which was used for making a decision regarding the significance of the terms and the model [39]. It was possible to determine those model components for which the *p*-Value is less than the established significance level by means of the results of the analysis of variance. A significance level of 5% was used during the analysis. The significance of the individual components of the coordinate measurement time model was determined based on the analysis of variance. Moreover, the results of the analysis of variance included information on the accuracy of the model in representing the measurement data. The *p*-Value parameter in the case of Lack-of-Fit indicates the insignificance of the lack of fit of the considered model. This means that the proposed model (1) can be regarded as sufficiently accurate.

**Figure 6:**
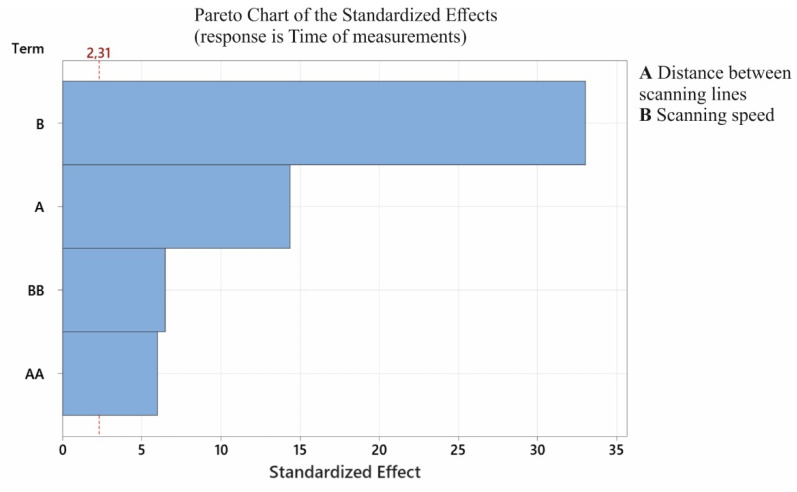
The graph of the significance of the components of the developed coordinate measurement time model.

Figure 6 presents a graph showing the importance of the individual components of the coordinate measurement time model. From the presented graph, it can be concluded that all components of the developed regression model were statistically significant.

**Figure 7:**
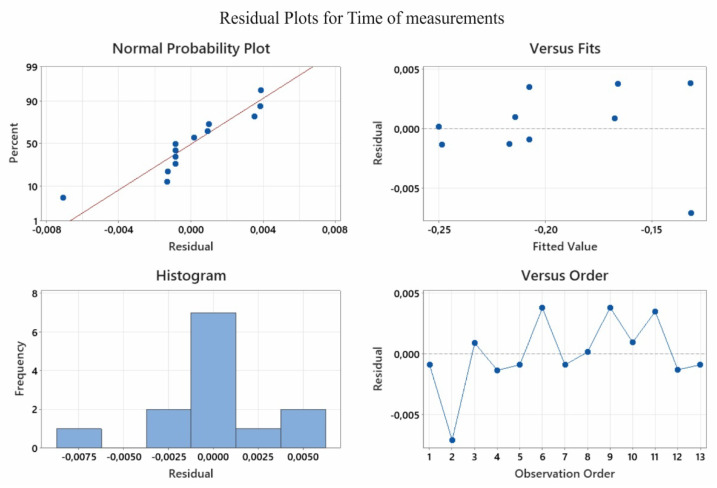
The residual plots obtained from the results of the experimental studies conducted.

**Figure 8:**
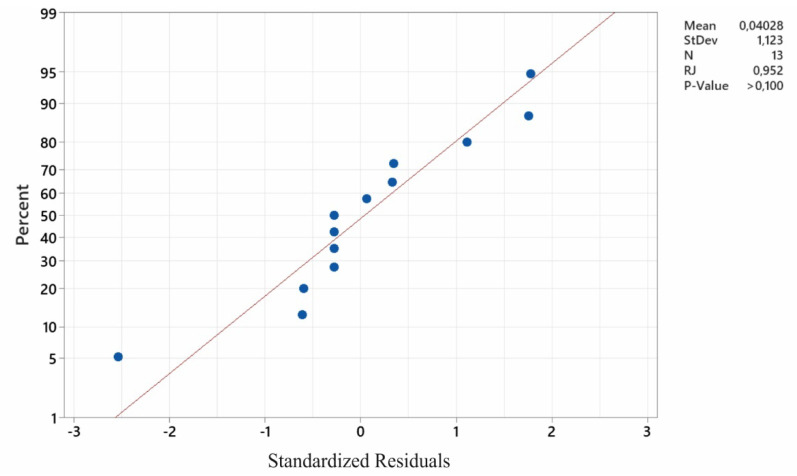
The results of the normality test.

Figure 7 presents the residual plots. They can be used for reference only. Inferring compliance with the normal distribution requires conducting a residual normality test. The results of the normality test are presented in Figure 8. The Ryan–Joiner test was applied. On the basis of the results presented in this graph, there are no grounds to reject the hypothesis of a normal distribution of the population from which the analysed sample was sourced. This means that the model is correctly constructed and calculated, and is not biased; i.e., it does not underestimate or overestimate the results.

**Figure 9:**
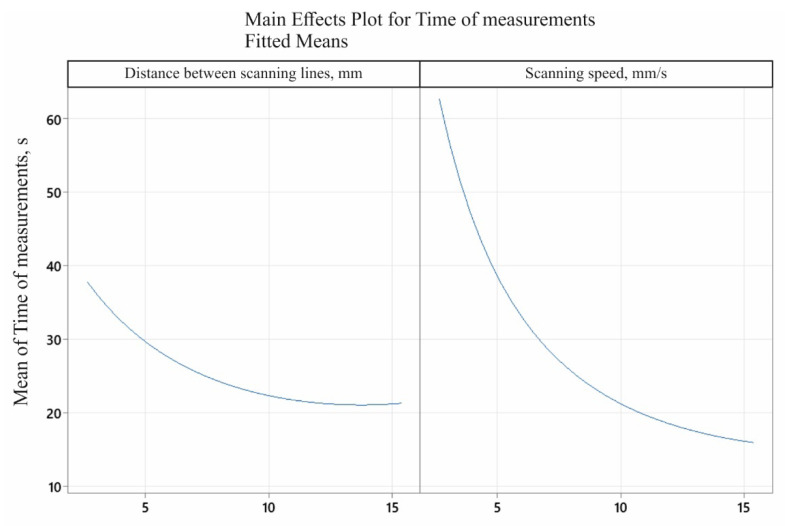
The influence of the input factors on the coordinate measurement time required to determine the flatness deviation.

Figure 9 shows the influence of the two analysed input factors (i.e., the distance between the measurement paths and the scanning speed of the measuring probe) on the output factor (i.e., the time needed to take contact measurements to determine the flatness deviation of the selected measured surface). The coordinate measurement time for the measurement characteristic under consideration decreased with an increase in both the distance between the measurement paths (up to approximately 14 mm) and the value of the measurement velocity. Increasing the distance between measurement paths generates a smaller number of measurement points distributed on the measured surface of the product.

**Figure 10:**
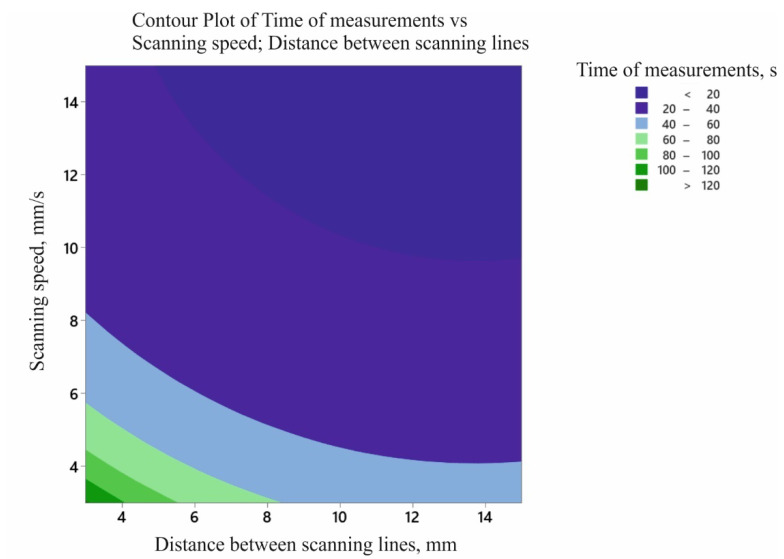
Contour plot of the contact coordinate measurement time model.

The contour plot of the contact coordinate measurement time model is shown in Figure 10. It is possible to determine the coordinate measurement time based on the adopted parameters of the measurement strategy. This, in turn, enables proper planning of the coordinate measurement process, taking into account not only the uncertainty of measurements, but also the time of their realisation.

**Figure 11:**
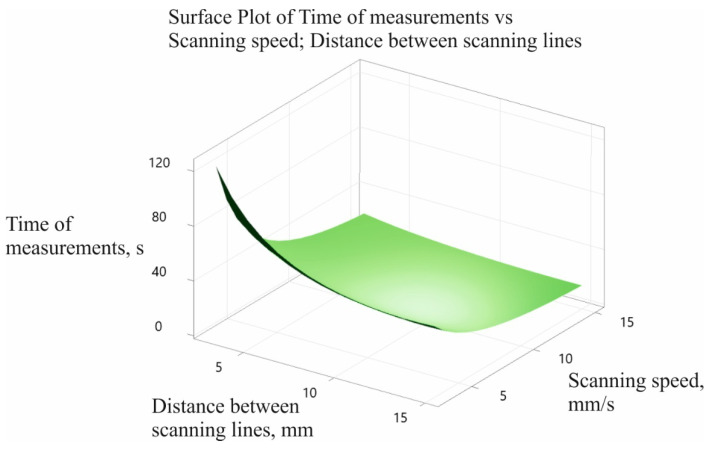
Graph of the contact coordinate measurement time model.

Figure 11 shows the final surface plot of the contact coordinate measurement time model.

However, when planning a strategy for coordinate measurements under industrial conditions, analysis of the graph presented in Figure 11 may be troublesome for people working in quality control departments and responsible for preparing the measurement program controlling a coordinate measuring machine. Therefore, it is necessary to implement a coordinate measurement time model developed for each measurement task into the selected metrological software, in order to facilitate the estimation of the measurement time. This implementation will help operators of coordinate measuring systems to automatically verify the duration of coordinate measurement processes on the basis of the adopted measurement parameters; for example, the scanning speed in the case of using scanning measuring probes.

## 4. Implementation of the Coordinate Measurement Time Model in the Metrology Software

**Figure 12:**
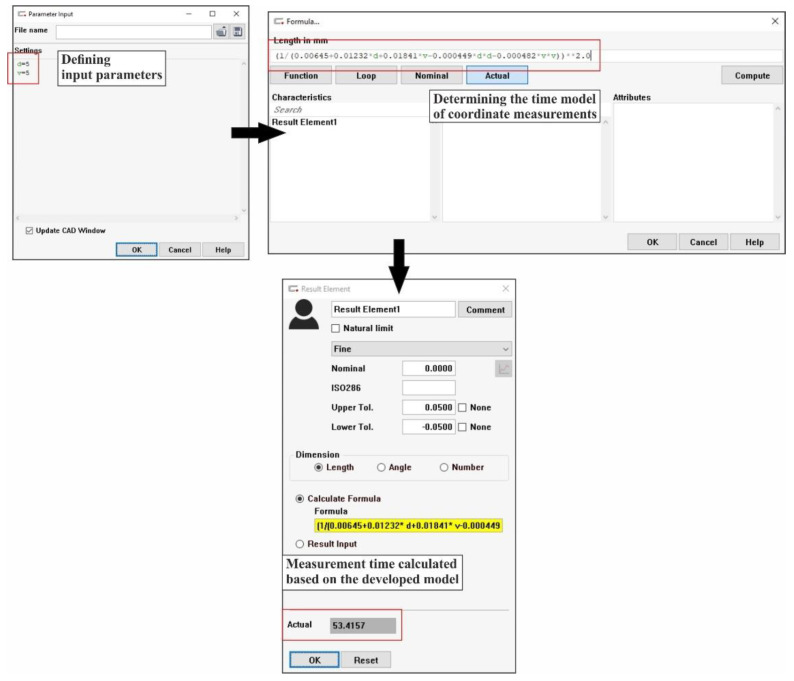
Implementation of the coordinate measurement time model in the selected metrology software cooperating with the ACCURA II coordinate measuring machine.

The coordinate measurement time model developed was implemented in the Calypso software cooperating with the applied CMM ACCURA II. This process was carried out using the PCM parametric programming module of the above-mentioned metrology software. In the first stage of implementation, two parameters corresponding to the input parameters (distance between measurement paths and scanning velocity), were created in the Calypso software. The values of the input parameters can be imported into Calypso through a text file. In the next stage, the equation of the contact coordinate measurement time model was determined. The last stage concerned the calculation of the time needed for the coordinate measurement process on the basis of the imported values of the input parameters (Figure 12). The calculated time corresponds to the value of the output parameter shown in Figure 10 and Figure 11.

## 5. Conclusions

The results of the experimental studies conducted confirmed that it is possible to estimate the time duration for coordinate measurements on the basis of the adopted measurement strategy. Knowing the duration of a given measurement task before starting actual measurements makes the implementation of effective coordinate measurements of products possible.

The use of response surface methodology enables the production of mathematical models of contact coordinate measurement times, which can be implemented in commercial inspection software packages cooperating with a coordinate measuring machine. The ability to implement the model in commonly applied metrology software makes it possible to use the model under industrial conditions.

The coordinate measurement time model, which represented the measurement data well, was obtained from experimental investigations. The coefficient of determination of the created model was equal to 99.42%. Moreover, the *p*-Value parameter in the case of Lack-of-Fit was greater than 5%, which indicates the insignificance of the lack of fit of the model (1).

A deficiency of the developed model is in obtaining correct measurement times when the distance between measurement paths is less than approximately 13–14 mm. However, the use of other types of models (e.g., linear), did not lead to obtaining satisfactory results for the Lack-of-Fit parameter.

The proposed coordinate measurement time model is correct for the measurement task that was under consideration in this study. However, it can be used in the case of measurements carried out with any coordinate measuring machine equipped with a scanning measuring probe. In the case of other measurement tasks, the proposed procedure (based on response surface methodology) leading to the determination of the coordinate measurement time model can easily be applied to other measurement characteristics and to products obtained by any manufacturing method (e.g., for measurement of a flatness deviation of a surface with holes). It only requires the use of a different measurement path for the measuring probe and modification of the input parameters.

Further investigations may lead to the development of other mathematical models that include various elements of measurement strategies applied to an inspection process conducted using coordinate measuring systems as input parameters to determine the accuracy of machining of products. For example, the presented methodology can be used in the case of an analysis of geometric deviations of products.

## Figures and Tables

**Table 1:** The experimental research plan.

StdOrder	RunOrder	PtType	Distance, mm	Scanning Speed, mm/s
13	1	0	9	9
1	2	1	4.76	4.76
2	3	1	13.24	4.76
8	4	−1	9	15
12	5	0	9	9
5	6	−1	3	9
10	7	0	9	9
4	8	1	13.24	13.24
7	9	−1	9	3
3	10	1	4.76	13.24
9	11	0	9	9
6	12	−1	15	9
11	13	0	9	9

**Table 2:** The coordinate measurement times taken for the adopted measurement strategies in order to determine a flatness deviation.

RunOrder	Time of Measurements, s
1	23
2	52
3	36
4	16
5	23
6	38
7	23
8	16
9	61
10	22
11	24
12	21
13	23

**Table 3:** The results of the analysis of variance.

Source	DF	Adj SS	Adj MS	F-Value	*p*-Value
Model	4	0.017098	0.004274	341.51	0.000
Linear	2	0.016232	0.008116	648.45	0.000
Distance	1	0.002591	0.002591	207.04	0.000
Scanning speed	1	0.013641	0.013641	1089.85	0.000
Square	2	0.000866	0.000433	34.58	0.000
Distance · Distance	1	0.000454	0.000454	36.28	0.000
Scanning speed · Scanning speed	1	0.000524	0.000524	41.87	0.000
Error	8	0.000100	0.000013		
Lack-of-Fit	4	0.000085	0.000021	5.49	0.064
Pure Error	4	0.000015	0.000004		
Total	12	0.017198			

## Data Availability

The data presented in this study are available on request from the author.
